# Mucosa-Associated Lymphoid Tissue Lymphoma Involving the Kidney and Renal Pelvis

**DOI:** 10.7759/cureus.15172

**Published:** 2021-05-22

**Authors:** Alexandre Gromicho, Débora Araújo, Vítor Oliveira, Ana Ribeiro, Luís Ferraz

**Affiliations:** 1 Urology Department, Hospital Central Funchal, Funchal, PRT; 2 Urology Department, Centro Hospitalar Vila Nova de Gaia/Espinho EPE, Vila Nova de Gaia, PRT; 3 Pathology Department, Centro Hospitalar Vila Nova de Gaia/Espinho EPE, Vila Nova de Gaia, PRT

**Keywords:** mucosa-associated lymphoid tissue (malt), lymphoma, kidney, nephroureterectomy, upper urinary tract

## Abstract

Mucosa-associated lymphoid tissue (MALT) lymphomas are a distinctive group of B-cell lymphomas. These lymphomas arise from various anatomic sites and are mainly seen in the gastrointestinal tract, but the primary involvement of the kidney is extremely rare. We report a case of a MALT lymphoma involving the kidney and the renal pelvis. A 56-year-old man presented with a history of hematuria and left flank pain. A computed tomography scan showed a marked tissue densification in the renal sinus, suggesting marked thickening of the urothelium, conditioning deformity of the renal pelvis and calyces. A cystoscopy confirmed a 2-cm papillary lesion on the left lateral aspect of the bladder. The patient underwent laparoscopic radical nephoureterectomy and transurethral bladder resection. The pathological diagnosis was MALT lymphoma in the kidney and urothelial carcinoma of the bladder. The patient was referred to a hematologist and was free of disease at 20 months of follow up without additional treatment.

## Introduction

First reported by Isaacson et al. in 1983 [1], extranodal marginal zone B-cell lymphoma of mucosa-associated lymphoid tissue (MALT), also known as MALT lymphoma, is a distinctive group of B-cell lymphomas [2,3]. They are one of the less aggressive lymphomas and often present as an indolent and localized disease [4]. MALT lymphomas arise from various anatomic sites and are mainly seen in the gastrointestinal tract, but the primary involvement of the kidney is extremely rare [5,6]. Currently, there are no conclusive recommendations for the management of renal MALT lymphoma [7,8]. We report a case of MALT lymphoma involving the kidney. Written informed consent was obtained from the patient.

## Case presentation

**Figure 1:**
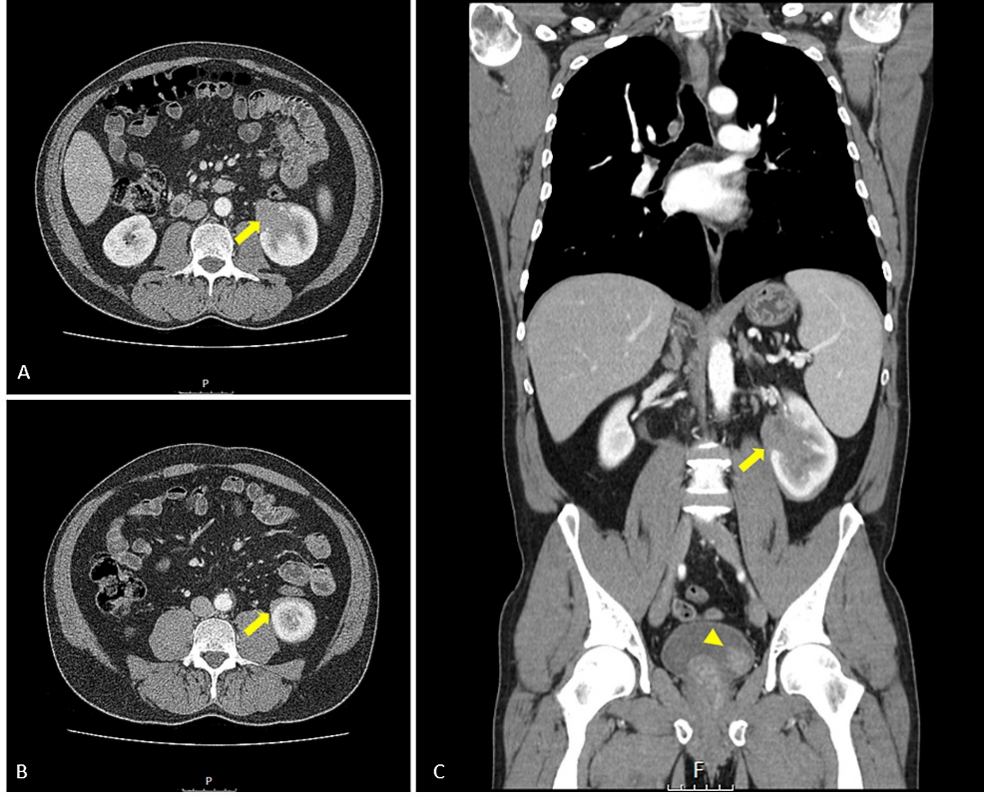
Axial (A and B) and coronal (C) CT scan Marked tissue densification in the renal sinus, suggesting marked thickening of the urothelium, conditioning deformity of the renal pelvis and calyces, particularly the middle and lower, with some heterogeneity of the renal parenchyma, highly suggestive of renal tumour (arrow); polypoid mass with 21 mm on the left lateral wall (arrowhead).

A 56-year-old man with a history of smoking was referred to our Urology department when the bladder and left kidney masses were detected by ultrasound during an evaluation for hematuria and left flank pain. Physical examination showed no remarkable findings. Regarding laboratory tests, complete blood cell and blood chemistries, including renal function tests, were normal. Urine analysis exhibited hematuria. Cystoscopy confirmed a 2-cm papillary lesion on the left lateral aspect of the bladder. A contrast-enhanced CT scan showed marked tissue densification in the renal sinus, suggesting marked thickening of the urothelium, conditioning deformity of the renal pelvis and calyces, particularly the middle and lower, with some heterogeneity of the renal parenchyma, highly suggestive of renal tumour (Figure 1).

**Figure 2:**
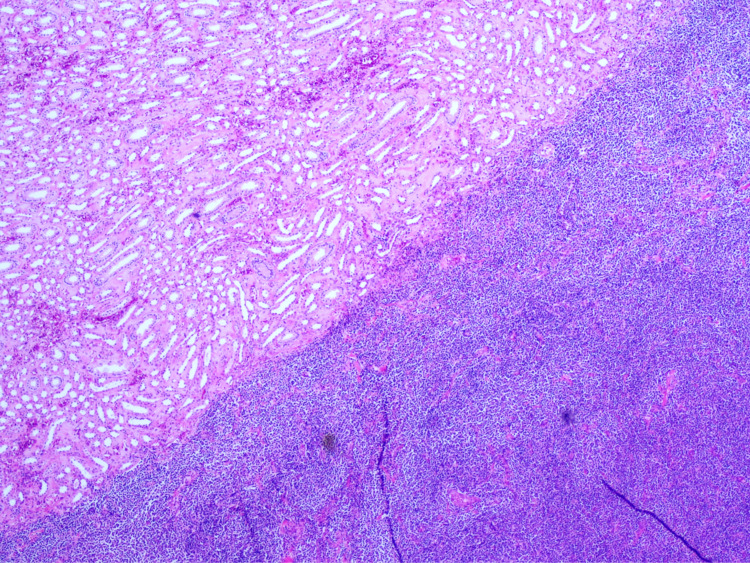
Histological analysis at low-power field On HE, at low power, it can be observed normal kidney parenchima (upper-left) and a diffuse monotonous lymphoid infiltrate (lower-right) that has replaced kidney tissue. It has no particular architecture. HE - hematoxylin and eosin

**Figure 3:**
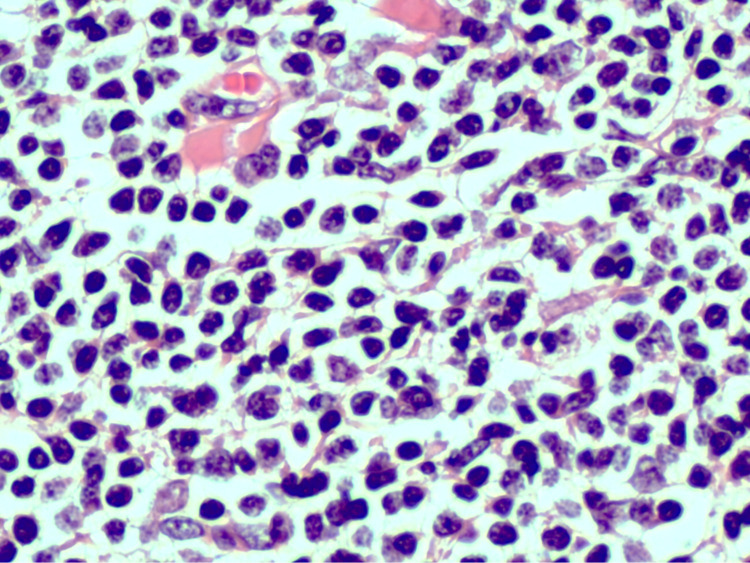
Histological analysis at high-power field At high power, the lymphoid infiltrate is composed of small lymphocytes with centrocytoid plasmacytoid morphology.

At this point, we suspected a urothelial cell carcinoma and the patient underwent laparoscopic left radical nephroureterectomy and transurethral resection (TUR) of the bladder. The pathology specimen contained a mass infiltrating the renal pelvis and parenchyma. On histological examination, atypical lymphoid proliferation was observed, showing plasmacytoid differentiation (Figures 2, 3).

**Figure 4:**
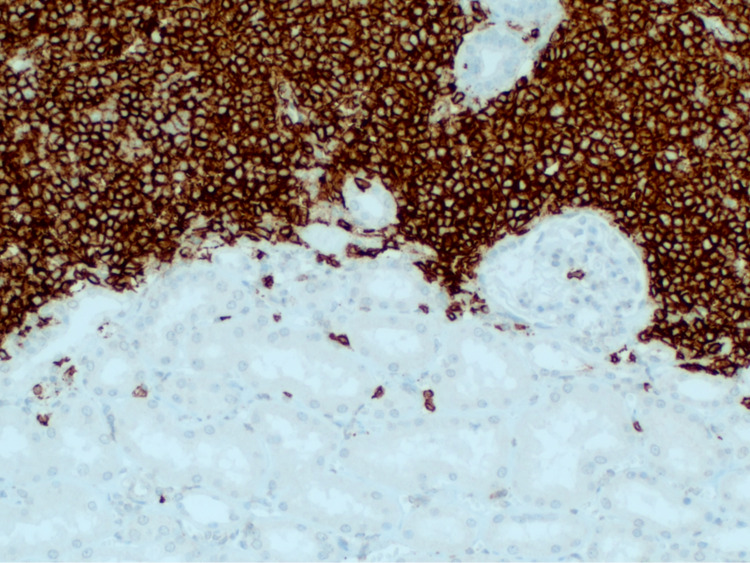
Flow cytometric immunophenotyping On immunohistochemistry, it is proven that the lymphoid infiltrate is composed mainly of B-lymphocytes (CD20+). It can also be appreciated as lymphoepithelial lesions in some kidney tubules. These cell only were positive for Bcl-2, other than CD20, and showed no expression for CD5, CD23, CD10, Bcl-6, and Cyclin D1 excluding other small B-cell lymphomas.

Flow cytometric immunophenotyping showed that the neoplasm was positive for immunoglobulin light chain kappa and for CD20, but negative for CD3, CD5, CD10, Bcl-6, Bcl-2, CD23, cyclin D1, and CD38 (Figure 4).

These findings supported a diagnosis of renal MALT lymphoma. No postoperative complications were recorded. The patient was referred to a hematologist and no further treatment was given. Furthermore, the pathology analysis of the bladder specimen revealed a high-grade urothelial cell carcinoma and the patient underwent intravesical bacillus Calmette-Guérin for one year. The patient was disease-free at 20 months of follow up.

## Discussion

Secondary renal involvement in non-Hodgkin’s lymphoma is common, but primary renal lymphoma is extremely rare [9,10]. MALT lymphomas arise from various anatomic sites, but the most frequent is the stomach. Others include the skin, salivary glands, ocular adnexa, intestines, and lung [3,10]. The kidneys are rarely involved [8,10-12].

These neoplasms arise at extranodal sites and are associated with chronic inflammation as a result of an infection or autoimmune disorder. This process is better established in the stomach, where MALT lymphomas are usually associated with Helicobacter pylori infection [3,5]. The etiology of renal MALT lymphoma is not well established since the kidney has no lymphoid tissue [12]. However, repetitive injury to renal lymphatics due to a chronic inflammatory process, such as chronic pyelonephritis, usually precedes the onset of MALT lymphoma [11]. Other disorders described in the literature as possible etiological factors are Sjögren’s syndrome, IgA nephropathy, membranoproliferative glomerulonephritis, Epstein-Barr virus, actinomycosis, sarcoidosis and systemic lupus erythematosus [5].

MALT lymphomas are often asymptomatic, being detected incidentally in radiological imaging studies. When symptomatic, flank pain, weight loss, hematuria, and rarely palpable mass can occur [7,13]. The diagnosis is challenging and crucial to distinguish from other neoplasms like renal cell carcinoma (RCC) and urothelial carcinoma [8]. The typical imaging pattern of MALT on contrast-enhanced CT is a large infiltrative renal tumour that often extends into the perinephric fat and retroperitoneum, with minimal contrast-enhanced and rarely vascular invasive [7,8,13]. On MRI, hypointense is on T2 and hypointense to isointense in T1, with minimal contrast enhancement [13]. These imaging studies are also important to exclude additional extranodal lesions. However, these findings are not usually sufficient to distinguish MALT lymphoma from other neoplasms, and the definitive diagnosis usually requires a biopsy. It is important to note that percutaneous biopsy is not recommended in suspicion of urothelial carcinoma because of the risk of tumour seeding [14]. Due to its differential diagnosis with the much more common RCC and urothelial carcinoma, and lack of specificity in most clinical and imagiologic findings, most diagnoses are made after radical nephrectomy [11]. The definitive diagnosis of MALT lymphoma is generally made based on histologic features and immunophenotype. The morphology is characterized by monocytoid B cells, small lymphocytes with abundant cytoplasm and lymphoepithelial lesions [7]. The immunohistochemical analysis is less specific and usually is negative for CD10 and cyclin D1 [9].

MALT lymphomas usually present as a localized disease with slow clinical progression [6]. However, transformation to high-grade lymphoma in the late course of the disease was reported in 8% of MALT lymphoma patients [15].

The therapeutic strategies for renal MALT lymphoma are controversial and there are no current established recommendations [7]. These neoplasms can be treated with chemotherapy, surgery or radiotherapy. When treated surgically in an early stage most patients do not receive additional treatment [8]. Chemotherapy remains the mainstay of treatment in the presence of systemic disease [16]. The prognosis is reported to be good with chemotherapy, surgery or radiotherapy. The five-year overall survival and cancer-specific mortality of patients with MALT lymphomas originated in the genitourinary tract are 75.6% and 12.4%, respectively [17].

In our case, the etiology could not be identified. There was no evidence of any prior infection or autoimmune disorder. The diagnosis was difficult and challenging. The presence of hematuria, pain, concurrent papillary bladder lesion and the absence of other lesions on CT scan, especially enlarged lymph nodes, raised the suspicion of urothelial carcinoma. Thus, the patient underwent radical nephroureterectomy and the definite diagnosis was established after histological specimen examination. As this lymphoma can be treated with chemotherapy, a preoperative biopsy could have confirmed the diagnosis and prevented an unnecessary nephrectomy. However, the suspicion of urothelial cancer was high, with synchronous lesions on the bladder and upper urinary tract, and no biopsy was performed. Afterwards, the patient was referred to a hematologist and no further treatment was given based on the chemotherapy adverse effects and the good prognosis of localized disease.

## Conclusions

In summary, we have described a case of a renal MALT lymphoma treated with minimally invasive surgery. Although it is a rare pathology, it should be considered in a differential diagnosis in cases of atypical renal mass. Early diagnosis and appropriate treatment provide a favorable outcome.
